# Cost-effectiveness analysis of PCR for the rapid diagnosis of pulmonary tuberculosis

**DOI:** 10.1186/1471-2334-9-216

**Published:** 2009-12-31

**Authors:** Luciene C Scherer, Rosa D Sperhacke, Antonio Ruffino-Netto, Maria LR Rossetti, Claudia Vater, Paul Klatser, Afrânio L Kritski

**Affiliations:** 1 Universidade Luterana do Brasil-ULBRA, Canoas/RS/Brazil; 2 Centro de Desenvolvimento Científico e Tecnológico, CDCT, Fundação Estadual de Produção e Pesquisa em Saúde, FEPPS/RS) Porto Alegre/RS/Brazil; 3 Programa Acadêmico de Tuberculose, Faculdade de Medicina/Complexo Hospitalar: HUCFF-IDT, Instituto de Saúde Coletiva/IESC/Universidade Federal do Rio de Janeiro - UFRJ, Rio de Janeiro, Brazil; 4 Royal Tropical Institute (KIT), KIT Biomedical Research, the Netherlands; 5 Faculdade de Medicina de Ribeirão Preto, Universidade de São Paulo, São Paulo, Brazil

## Abstract

**Background:**

Tuberculosis is one of the most prominent health problems in the world, causing 1.75 million deaths each year. Rapid clinical diagnosis is important in patients who have co-morbidities such as Human Immunodeficiency Virus (HIV) infection. Direct microscopy has low sensitivity and culture takes 3 to 6 weeks [1-3]. Therefore, new tools for TB diagnosis are necessary, especially in health settings with a high prevalence of HIV/TB co-infection.

**Methods:**

In a public reference TB/HIV hospital in Brazil, we compared the cost-effectiveness of diagnostic strategies for diagnosis of pulmonary TB: Acid fast bacilli smear microscopy by Ziehl-Neelsen staining (AFB smear) plus culture and AFB smear plus colorimetric test (PCR dot-blot).

From May 2003 to May 2004, sputum was collected consecutively from PTB suspects attending the Parthenon Reference Hospital. Sputum samples were examined by AFB smear, culture, and PCR dot-blot. The gold standard was a positive culture combined with the definition of clinical PTB. Cost analysis included health services and patient costs.

**Results:**

The AFB smear plus PCR dot-blot require the lowest laboratory investment for equipment (US$ 20,000). The total screening costs are 3.8 times for AFB smear plus culture versus for AFB smear plus PCR dot blot costs (US$ 5,635,760 versus US$ 1,498, 660). Costs per correctly diagnosed case were US$ 50,773 and US$ 13,749 for AFB smear plus culture and AFB smear plus PCR dot-blot, respectively. AFB smear plus PCR dot-blot was more cost-effective than AFB smear plus culture, when the cost of treating all correctly diagnosed cases was considered. The cost of returning patients, which are not treated due to a negative result, to the health service, was higher in AFB smear plus culture than for AFB smear plus PCR dot-blot, US$ 374,778,045 and US$ 110,849,055, respectively.

**Conclusion:**

AFB smear associated with PCR dot-blot associated has the potential to be a cost-effective tool in the fight against PTB for patients attended in the TB/HIV reference hospital.

## Background

Tuberculosis is one of the most important health problems in the world, causing 1.75 million deaths each year, in 2007. Rapid clinical diagnosis is more challenging in patients who have co-morbidities, such as Human Immunodeficiency Virus (HIV) infection. Direct microscopy has low sensitivity and culture takes 3 to 6 weeks [1-3]. Diagnostic testing for tuberculosis has remained unchanged for nearly a century, but newer technologies hold promise for a revolution in tuberculosis diagnostics. Tests such as the nucleic acid amplification assays allow more rapid and accurate diagnosis of pulmonary and extrapulmonary tuberculosis. The appropriate and affordable use of any of these tests depends on the setting in which they are employed [4,5]. New tools for TB diagnosis are necessary, especially in health settings with a high prevalence of HIV/TB co-infection.

In developing countries, *in house *polymerase chain reaction (PCR) based on amplifying the IS*6110 *insertion element can be used for the amplification of *Mycobacterium tuberculosis *(MTB) DNA and offers the potential of a sensitive, specific and rapid diagnostic for ruling out or considering pulmonary tuberculosis (PTB) [6-9].

The majority of previous studies have evaluated *in house *and automated PCR and reported PCR sensitivities ranging from 77% to 95% and PCR specificities of 95% in smear-positive specimens, using culture as the gold standard and clinical criteria only to evaluate the discrepant results. Moreover, the PCR tests were evaluated separately, in contrast to clinical practice, where tests for diagnosis are required in association [6,10,11]. Many prior studies have observed that routine clinical use of PCR may be difficult due to its high cost, particularly if PCR is used by itself, and emphasize the importance of clinical utility and cost effectiveness analysis for these tests as a better argument for making such a decision [9-15]. For regions with a high burden of TB and HIV, which urgently needed new strategies for TB control, there are scarce data on cost-effectiveness analysis of the PCR technique for TB diagnosis [11].

In the present study, in a hospital setting with a high burden of TB and HIV, we investigated the cost-effectiveness of a home-made colorimetric PCR (PCR dot-blot) to diagnose TB using expectorated sputum from patients suspected of having PTB, in parallel with direct microscopy by Ziehl-Neelsen staining for PTB diagnosis, using the combination of positive culture with the clinical definition of PTB as the gold standard.

## Methods

### Setting and patient selection

Consecutive adults suspected of having PTB, referred to the TB and HIV Reference Center, Parthenon Reference Hospital (PRH) in Porto Alegre City, capital of Rio Grande do Sul, State of Brazil, were studied prospectively, from May 2003 to May 2004. Eligible patients were those: (1) who reported more than 3 weeks of coughing; Patients were excluded from the study if any of the following conditions were met: (1) culture was contaminated; (2) when expectorated sputum was not obtained (3) laboratory or clinical data did not fulfill the PTB definition; (4) written informed consent was not obtained from the study participant. All clinical samples were sent to the Laboratory of the State of RS, State Foundation for Research in Health, Porto Alegre/RS/Brazil, (FEEPS/LACEN/RS) for laboratory analysis. This study was approved by the Institutional Review Boards of FEPPS/RS (n. 01/2002).

Suspects of PTB, after signing their written informed consent, underwent a validated questionnaire with questions regarding demographic variables and clinical history (e.g.: smoking, alcohol abuse, HIV infection/AIDS)[16]. Chest radiographs and physical examination were performed by a respiratory specialist using a standardized form. Laboratory technicians and respiratory specialists were blinded for the results of cultures and PCR dot-blot, and for the chest radiographs results and clinical predictors, respectively. HIV testing by ELISA (GenScreen HIV Plus^® ^BioRad) was performed, using Western blot (Genelabs^® ^Diagnostics) as a confirmatory test.

### Case definition

PTB cases were defined as those with a positive culture for MTB in the respiratory specimen or those with clinical and radiological improvement after six months of solely anti-TB treatment, as judged by three different chest physicians in a blinded review, not involved in this study [17]. Not-PTB was considered in patients whose acid-fast smear and culture for MTB were negative and who had no chest radiographic changes after six months of follow-up. PCR results were not available for routine care or for the panel of experts.

Gold standard criteria for PTB final diagnosis included all PTB cases, confirmed or not by culture.

### Routine laboratory process and performance evaluation

All sputum specimens were processed at the Public Reference Laboratory. All sputum specimens were tested by the Ziehl-Neelsen method, cultured in Löwenstein Jensen and identified according to Kubica's method [18].

The presence of the amplified fragment derived from the *IS*6110 insertion element sequence in positive PCRs was checked by electrophoresis with 2% agarose gel, stained with ethidium bromide, and visualized under ultraviolet light [10]. The positive and negative controls were included in electrophoresis analysis.

The PCR colorimetric dot-blot assay was performed as previously published [10]. Briefly, the biotinylated PCR products were transferred to a nylon membrane and hybridization was performed with a specific probe. The detection of hybridization was performed using a conjugated streptavidin-alkaline phosphatase probe. The positive reaction was obtained by adding BCIP and NBT (5-bromo-4-chloro-3-indoyl phosphate and nitro blue tetrazolium). The positive and negative controls were included for each set of PCR. To detect specimen inhibitors in negative results, a tube of PCR mix for each specimen was spiked with purified DNA target. All PCR tests with discrepancies in results were tested in duplicate.

### Costs

The cost components for each procedure included costs incurred by the patient, laboratory costs, drugs, consumables and equipment costs. Number and level of staff screening for TB in the hospital were considered as the same in all strategies. Clinical, radiological and laboratory staff costs were calculated from the salary base of Rio Grande do Sul State of Brazil. For each procedure, costs were attributed based on procedure costs of the Brazilian Public Health System. For PCR, capital costs included the cost of the thermocycler, reader and centrifuge. Running costs (material costs used for each 1000 tests evaluated) included all laboratory materials used in procedures. All costs were expressed in US$, using an exchange rate of US$ 1 = 3 R$ (REAIS), the average exchange rate from 2003 to 2004. In the treatment costs, costs were evaluated related to the treatment of inpatients and outpatients. To estimate the values that are spent by the public health system of Brazil with the monitoring and control of TB in a hospital and an outpatient unit, we simulated two different scenarios: a) TB cases diagnosed in hospital wards (inpatients), b) TB cases diagnosed in outpatient environment (outpatients).

The number of days considered to calculate the costs related to the treatment of inpatients were considered as the same days that were spent on each laboratory procedure. It was hypothesized that the time to detect *Mycobacterium tuberculosis *in sputum culture from patients with pulmonary tuberculosis may be a better indicator for the duration of time of hospitalization[19]. The time to detect *M. tuberculosis *in the culture was 30 days in this study. This cohort is the same as previous published by our group [20]. The median time to reveal growth of MTB was 30 days (interquartile range [IQR] 30 to 45) for smear/culture and the median time for detection of MTB by PCR was 3.32 days (IQR 3.0 to 3.75), respectively (p < 0.01). This value was used as the standard at which release from isolation could be permitted [20]

The time spent on each laboratory procedure to provide access to the result of the laboratory technique was assumed to be 3 days for AFB smear plus *in house *PCR (PCR dot blot), and 30 days for AFB smear plus culture. The number of days considered to calculate costs was the same as those spent on each laboratory procedure. The number of days considered to calculate the cost of patient travel costs was assumed to be 2 days for AFB smear plus *in house *PCR (PCR dot blot), and 2 days for AFB smear plus culture.

Total treatment included clinical officer and hospital costs, assuming cost per pill, to be US$ 0.22, using 3 pills per day, during 180 days; hospital room costs, US$ 4.16/day; costs with salary of clinical staff and clinical consultation, US$2.52 per patient and clinical nursing consultation, US$2.52 per patient.

Assuming that, during the treatment (6 months), in ambulatory situation, 6 AFB smear test, 6 chest radiographs, 6 consult of nurse and 2 consult of clinical were performed, we used this parameters to estimated the costs of ambulatory following the Brazilian recommendations for treatment [21].

Assuming that, during the hospitalization (30 days), 4 AFB smear tests, 4 chest radiographs, 30 nurse and physician consultations were performed, we used these parameters to estimated the costs of inpatient assistance in hospital, following the Brazilian recommendations for treatment [21]. Staff salaries for the physician, nurse and radiologist were considered to be US$6,400 per year, and for the chest radiograph technician, the salary was US$2,860 per year. The work days were considered 20 days for all staff.

The days of admission to the hospital were considered to be the same number of days spent on each laboratory procedure. All estimated costs reflect an estimate of the public health system of Brazil expenses with the monitoring and control of TB.

The costs were expressed per 1000 suspects, according to the specific bibliographic references for economic analyses, thus, allowing the best decision for investment to be made [22].

### Cost effectiveness

A decision analytic model was developed using the Excel 7.0 software to estimate the costs of the routine diagnostic procedures for diagnosis of PTB using sputum specimens: 1) AFB smear used with culture, and 2) AFB smear used with PCR dot blot. We evaluated the the cost effectiveness ratio of cost per case correctly diagnosed of TB, of cost per case accurately diagnosed and treated, including treatment of incorrectly diagnosed cases (e.g. false positive patients), and of cost per case incorrectly diagnosed and not treated (e.g. false negative patients). We assumed that, 10 false negative patients may transmit *M. tuberculosis *to 100 individuals, and active TB is expected to occur in 5% of those infected. In this study, the rate of TB in false negative patients was 13.3% for AFB smear plus Culture strategy and 14.8% for AFB smear plus PCR dot-blot strategy. Assuming the diagnosis of 1,000 TB cases, additional active TB cases is expected to occur with AFB smear plus culture and AFB smear plus PCR dot strategy in 66,5 and 74,0, respectively. We calculated the costs of missing false negatives and compare the costs of each strategy [22].

A sensitivity analysis was performed to assess the effect of the various parameters (TB prevalence, sensitivity, specificity, and variable costs) on the conclusions.

## Results

A total of 277 TB suspects, all with known HIV test results, were enrolled. Prevalence of PTB was 46.2% (128/277) overall and 54.0% (40/74) among HIV infected subjects. Test performance and cost-effectiveness analyses were not stratified by HIV status.

### Test performance

Table 1 shows the yield and performance of each combined procedure in detecting a PTB case. The positive (LR+) and negative (LR-) likelihood ratios were 89 and 0.13 for AFB smear plus culture, and 5.31 and 0.18 for AFB smear plus PCR-dot-blot, respectively. The inhibition of two *in house *PCR was 1.9%. Twenty-three specimens presented less than 50 CFU in culture, which is below the detection limit of the PCR. These specimens were included in the analysis.

**Table 1:** Sensitivities, specificities and likelihood ratios of AFB smear plus Culture and AFB smear plus PCR dot-Blot

Laboratory Results and Performance of methods^a^		Total number of suspects N = 277			
		TB N = 128		Not-TB N = 149	
**Performance of AFB smear plus Culture**	**Positive**	111		1	
	**Negative**	17		148	

		**SE**	**SP**	**LR+**	**LR-**
		87%	99%	89	0.13

**Performance of AFB smear plus PCR dot-blot**	**Positive**	109		22	
	**Negative**	19		127	

		**SE**	**SP**	**LR+**	**LR-**
		85%	85%	5.31	0.18

SE: Sensitivity, SP: Specificity, LR+: Positive Likelihood Ratio, LR-: Negative Likelihood Ratio. ^a^The performance of tests was calculated using specific formulae utilized by parallel tests: sensitivity (SE) of AFB smear with culture: (SE_zn _+ SE_culture_) - (SE_zn _× SE_culture_), specificity (SP) of AFB smear with culture: SP_zn.X _SP_culture_, predictive values (PV) and likelihood ratio (LR), according to literature [32].

### Costs

Table 2A shows the costs at the health service level and Table 2B shows costs due to laboratory investment. The AFB smear plus PCR dot-blot require the lowest laboratory investment for equipment (US$ 20,000). Table 2C shows costs incurred by patients. The AFB smear plus PCR dot-blot require the lowest costs incurred by patients (US$ 34.0 per patient).

**Table 2:** Costs in US$ of TB diagnosis in Brazil

A. Health service costs				
	**Staff Number**	**Salaries of all staff per year** **(US$)**	**Staff Cost per day** **(US$)**	**Time spent until access to result** **(days)**

**AFB smear plus Culture**	2	9,260	39	30

**AFB smear plus PCR-dot-blot**	2	9,260	39	3

**B. Laboratory costs**				

	**Equipment** **(US$)**	**Annualization Years**	**Running costs per 1000 suspects** **(US$)**	**Running costs per examination** **(US$)**

**AFB smear plus Culture^a^**	22,667	5	12,333	12.33

**AFB smear plus PCR-dot-blot^b^**	20,000	5	12,833	12.83

**C. Estimated costs incurred by patients, including costs for travel, food and income loss^d^**				

	**AFB smear plus Culture (US$)** **(outpatients and inpatients)**		**AFB smear plus PCR dot-blot (US$) (inpatients and outpatients)**	

**Travel**	1,000		1,000	

**Food**	3,000		3,000	

**Income Loss^c^**	186,800		30,000	

**Total patients Cost**	190,800		34,000	

^a ^Microscopic and Laminar Flow Cabinets,;

^b^Thermocycler reader, Centrifuge and Laminar Flow Cabinets. Other equipments were not included,

^c ^Income loss of patients was calculated from monthly salary base of Brazil (US$117) and was based on proportional days spent by patients until access to the result of each laboratory procedure. Patient costs were estimated using the average of two visits to the laboratory for the PCR procedure and for AFB smear and culture procedures for outpatients; Travel cost was considered as US$ 0.8 (one way by bus). Food was considered as US$3.3 per meal. Base salary in Brazil was considered (US$5. 83 per day/20 days of the work). For inpatients was considered just income loss; Staff costs in the laboratory were based on proportional days spent on each laboratory procedure;Costs of consumables and equipment were provided by the program as well as by the manufacturer. We annualized the capital cost of the equipment for 5 years, according to the literature [25]. PCR capital costs included the cost of the thermocycler, reader and centrifuge. Building costs were not included. Opportunity costs were not applicable.

Table 3 shows the total cost of screening 1000 suspects. When total screening costs were considered, AFB smear plus PCR dot blot costs were 3.8 times lower than AFB smear plus Culture (US$ 1,498,660 versus US$ 5,635,760).

**Table 3:** Costs (in US$) of screening 1000 TB suspects, comparing AFB smear plus Culture and AFB smear plus PCR dot-blot

	AFB smear plus Culture	AFB smear plus PCR dot-blot
Total cases TB	128	128
**3. A Health Service costs**		

**Labor costs^a^**		

**Laboratory Costs**	3,283	13,067
**Investment costs**	123	194
**Running costs**	12,333	12,833

**Treatment costs**		

**Treatment costs (ambulatory/outpatients)**	1,589	849

**Treatment costs (hospitalization/inpatients)**	2,686	487

**Treatment costs (outpatients and inpatients)**	4,275	1,336

		

**Diagnostic Service costs per day^a^**		

**Cost staff per time spent in each laboratory procedure**	1,158	116
		

**3. B1. Patient cost (ambulatory/outpatients)^d^**		

**Travel**	1	1

**Food**	3	3

**Income Loss**	12	12

**3. B2. Patient cost (hospital/inpatients)^d^**		

**Travel**	0	0
**Food**	0	0
**Income Loss**	174.8	18

**3. C Total costs for 1000 TB suspects**		

**Total Patient costs**	190,800	34,000

**Total Health Service costs**	5,444,960	1,464,660

**Total Screening costs**	5,635,760	1,498,660

^a ^For each procedure, costs were attributed based on procedure costs of the Brazilian Public Health System (US$ 1.4 for AFB smear and US$ 1.9 for Culture) and from CDCT/FEPPS (US$ 11.7 for PCR dot-blot), assuming investment laboratory equipment for 5 years; ^b^Staff salary was considered; for laboratory technician, US$2,860 per year; for Laboratory technologist, US$6,400 per year. Staff costs in the laboratory were based on proportional days spent on each laboratory procedure; Staff salary was considered for clinical physician, nurse and radiologist; US$6,400 per year; for the X-RAY technician, salary was US$2,860 per year. ^c^The days of hospitalization were considered as the same as the days spent on each laboratory procedure. The time spent on each laboratory procedure until access to the result of the laboratory technique was assumed to be 3 days for AFB smear plus *in house *PCR (PCR dot blot), and 30 days for AFB smear plus Culture. Total treatment included clinical officer and hospital costs, assuming US$ 0.22 cost per pill, using 3 pills for day, during 180 days; hospital room costs, US$ 4.16/day; costs of salary of staff clinical; clinical consultation cost, US$2.52 per patient; clinical nursing consultation, US$2.52 per patient. Assuming that during the treatment of inpatients (4 months) 4 AFB smear and 4 chest radiograph were performed, and during the treatment of outpatients (6 months) 6 AFB smear and 6 chest radiograph were performed, following the Brazilian recommendations for treatment [21];

^d ^Travel for AFB smear strategies was considered as 2 days for AFB smear plus Culture strategy; and 2 days for AFB smear plus PCR dot-blot. Food and income loss for AFB smear strategies was considered as 30 days for AFB smear plus Culture strategy; and 3 days for AFB smear plus PCR dot-blot The health service costs analysis was based on processing 50 AFB smear slides, 86 samples for each *in house *PCR and 14 cultures per day. AFB smear plus Culture and *in house *PCR were performed by two trained staff, respectively. Costs of chest physicians were considered the same for all strategies. Running costs were calculated from investments required to examine 1000 smears.

When considering total cost (in US$) related to the treatment (outpatients) per strategy, AFB smear plus PCR dot-blot costs were 1.8 times lower than AFB smear plus Culture (US$ 849 versus US$ 1,589). When considering the cost related to the treatment of inpatients, the total cost for AFB smear plus PCR dot-blot was 5.5 times lower than AFB smear plus Culture strategy (US$ 487 versus US$ 2,686). When considering the cost related to the treatment of outpatients and inpatients, the total cost for AFB smear plus PCR dot-blot was 3.2 times lower than the AFB smear plus Culture strategy (US$ 1,336 versus US$ 4,275).

Table 4 shows the cost-effectiveness of screening 1000 PTB suspects, comparing AFB smear plus culture and AFB smear plus PCR dot-blot. AFB smear plus PCR dot-blot was less costly than AFB smear plus culture. When the costs of treating all accurately diagnosed cases were considered, the cost-effectiveness was lower for AFB smear plus PCR dot-blot (US$ 13,749) than the AFB smear plus culture strategy (US$ 50,773). However, the cost of treatment of false positives cases was less cost-effective for the AFB smear plus PCR dot blot strategy than the AFB smear plus culture strategy (US$ 18,674 versus US$ 1,589) for outpatients. The cost of treatment of false positives cases was less cost-effective for the AFB smear plus PCR dot blot strategy than the AFB smear plus culture strategy (US$ 10,715 versus US$ 2,686) for inpatients. The cost of treatment of false negatives cases was more cost-effective for the AFB smear plus PCR dot blot strategy than the AFB smear plus culture strategy (US$ 25,382 versus US$ 72,675).

**Table 4:** Costs (in US$) and Cost-effectiveness (in US$) of screening 1000 TB suspects, comparing AFB smear plus Culture and AFB smear plus PCR dot-blot.

	AFB smear plus Culture	AFB smear plus PCR dot-blot
**Total number of accurately diagnosed cases of TB**	111	109

**Total number correctly diagnosed cases of non TB**	148	127

**False positives**	1	22

**False negatives**	17	19

**Estimate of false negatives in 1000 TB suspects**	133 (13.3%)	148 (14.8%)

**Estimate of cases of TB (5%) using the estimative of transmission of false negatives in 1000 TB suspects**	66,5	74

**4. A Total Costs**	(US$)	(US$)

**Total Health Service Costs**	5,444,960	1,464,660

**Total Patient Costs**	190,800	34,000

**Total Screening Costs**	5,635,760	1,498,660

**Total Screening Costs per patient**	**5,635**	**1,498**

**4. B Cost-effectiveness of screening of 1000 suspects**		

**Cost per accurately diagnosed case of TB**	50,773	13,749

**Cost per accurately diagnosed case of TB, considering only health service costs**	49,053	13,437

**Cost per accurately diagnosed case of TB, considering only patient cost**	1,720	312

**Cost of treating all correctly diagnosed TB cases (true positives cases) (ambulatory/outpatients)**	176,338	92,523

**Cost of treating all correctly diagnosed TB cases (true positives cases)** **(hospital/inpatients)**	298,187	53,087

**Cost of treating all falsely diagnosed TB cases (false positives cases) (ambulatory/outpatients)**	1,589	18,674

**Cost of treating all falsely diagnosed TB cases (false positives cases) (hospital/inpatients)**	2,686	10,715

**Cost per case of TB correctly diagnosed and case of TB falsely diagnosed and treated (true positives and false positives)^a^**	50,319	11,440

**Cost of treatment of false negatives cases of TB (Treatment costs vs false negative cases)**	72,675	25,382

**Cost per case of non-TB correctly and incorrectly diagnosed and not treated** **(true negatives and false negatives)**	34,156	10,260

**Cost of return of all false negatives to the health service (Estimate of cases of TB (5%) using the estimative of transmission of false negatives in 1000 TB suspects vs Total Screening Costs^b^**	374,778,045	110,900,055

^a ^including treatment of falsely diagnosed cases, ^b ^assuming that one false negative transmits TB to 10 individuals and these 5% have TB and the cost-effectiveness was expressed for 1000 suspects [35]

Considering the aim of this study was to compare the costs of AFB smear plus culture and AFB smear plus PCR dot blot strategies, Table 5 presents a sensitivity analysis to compare the cost-effectiveness ratio of the two strategies, adjusting for different features. The ratio of cost-effectiveness was 3.7 in most adjustments. Considering the high prevalence of TB (60%), the AFB smear plus PCR dot-blot was more cost effective than the current situation (prevalence, 46%). Considering low prevalence of TB (10%), the PCR dot-blot was less cost effective than the *status quo*, but the costs decreased with the increase in TB prevalence. If the running costs of PCR dot-blot were reduced by 22%, the AFB smear plus PCR dot blot was more cost effective than the AFB smear plus culture strategy.

**Table 5:** Sensitivity analysis (in US$) based on screening of 1000 suspects, comparing AFB smear plus Culture, and AFB smear plus PCR dot-blot

			Cost per accurately diagnosed case of TB			Cost per case accurately diagnosed and treated, including treatment of falsely diagnosed cases			Cost per case incorrectly diagnosed and not treated**		
**Component**	**Current ** **situation**	**Adjustment**	**AFB smear ** **plus Culture**	**AFB ** **smear plus ** **PCR ** **dot-blot**	**Ratio**	**AFB smear ** **plus Culture**	**AFB ** **smear ** **plus ** **PCR ** **dot-blot**	**Ratio**	**AFB smear ** **plus Culture**	**AFB smear ** **plus PCR ** **dot-blot**	**Ratio**

**TB prevalence**	**46%**	**No adjustment**	50,773	13,749	3.7	55,501	15,190	3.6	22,045,767,	5,836,887	3.8
		**10%**	233,554	63,246	3.7	255,304	69,874	3.6	124,926,015	27,725,214	4.5
		**20%**	116,777	31,623	3.7	127,652	34,937	3.6	20,821,002 $	3,960,745	5.3
		**40%**	58,389	15,812	3.7	63,826	17,469	3.6	9,609,693	1,980,372	4.9
		**60%**	38,926	10,541	3.7	42,551	11,646	3.6	505,773	94,303	5.4
**Sensitivity ** **PCR dot-blot**	**85%**	**95%**	46,577	12,386	3.7	50,914	13,683	3.7	3,097,339	916,536	3.4
**Specificity ** **PCR dot-blot**	**85%**	**95%**	39,688	10,554	3.7	43,384	11,659	3.7	2,639,282	780,992	3.4
**PCR dot-blot ** running costs*	**12,833**	**10,000**	50,772	13,723	3.7	55,501	15,164	3.7	20,045,767	5,825,852	3.8

* assuming that PCR dot-blot running costs could be decreased by 22%, ** assuming that one false negative transmits TB to 10 individuals and these 5% have TB and the cost-effectiveness was expressed for 1000 suspects.

## Discussion

The strategy of comparing rapid techniques, such as PCR, to standard techniques, such as AFB smear/culture could improve the quality of diagnosis and reduce delayed identification of mycobacterial infections. This is especially crucial among hospitalized PTB suspects, in which the atypical clinical presentation and mortality rate are more frequent, and where it is sometimes difficult to obtain multiple respiratory specimens [11,23]. Additionally, AFB smear alone cannot distinguish TB from mycobacteria other than TB [24]. In this study, in a setting with a high prevalence of TB/HIV (54.0%), we evaluated sensitivity, specificity, likelihood ratios, predictive values and compared the cost-effectiveness of the strategies; AFB smear plus culture and AFB smear plus PCR dot-blot for diagnosis of PTB [25].

Although the AFB smear plus culture strategy presented the highest performance for PTB diagnosis, AFB smear plus PCR dot blot strategy had a similar sensitivity (85%) and a similar negative low likelihood ratio (0.18) to those of AFB smear plus culture (87%, 0.13), respectively, suggesting that this strategy may offer improvement for ruling out TB diagnosis for pulmonary TB suspects, including HIV infected individuals, where it is critical to initiate prompt, specific treatment and a delayed diagnosis can be lethal. Additionally, this strategy could reduce the risk of dissemination and spread to other hospitalized patients and health care personnel [2,26].

The specificity of AFB smear plus PCR dot-blot (85%) was similar to a series described (84% to 87%) in developing countries, also using automated nucleic acid amplification tests (NAA), and lower than that described (>95%) in industrialized countries [6,23,27,28]

The low sensitivities found in AFB smear plus PCR dot-blot (85%) may be due to the presence of inhibitors that remain in the specimen after the extraction procedure, or a small number of mycobacteria that are unequally distributed in the test suspension or that exist below the detection limit of both *in house *PCR tests (PCR dot blot) (50 CFU)[10]. With regard to false negative results, 57.9% (11/19) for PCR dot-blot was below the detection limit of the amplification test. The proportion of inhibitors was 1.9% for the *in house *PCR, similar to those used in NAA tests (0.85% to 22.7%) [12,28]. The low copy numbers of IS*6110 *(insertion element) in MTB, are reported to be a factor in decreased sensitivity, although this has not been reported in Brazil[20].

To make rational decisions about the implementation of NAA in the medical routine, cost-effectiveness studies are essential [29-31]. Such studies provide insight into the composition of different cost components, which may be the most important factor from the patient and the health service's perspectives [32]. Here, we compared the cost-effectiveness of AFB smear with *in house *PCR and with culture on the first sputum specimen collection, including staff costs, using culture and clinical evaluation as the gold standard, in contrast to the cost-effectiveness analysis described by van Cleef et al in a reference ambulatory clinic in Kenya, where only culture for mycobacteria was used as the gold standard [11,33].

In this study, we used the criteria that a strategy for ruling out of TB diagnosis is cost-effective when it is less costly and when it is at least as effective as the AFB smear plus culture strategy. We found that the use of AFB smear plus PCR dot-blot was less cost-effective per case correctly diagnosed, than the cost-effectiveness described using the decision model for automated PCR (Roche Mycobacterium Tuberculosis Amplicor PCR system), probably due to the lower accuracy observed with *in house *PCR techniques [11,33]. The cost-effectiveness of AFB smear plus PCR dot-blot depends strongly on sensitivity and specificity of tests. Others factors are important, such as the costs of the health service and also patient costs, the later usually not included in the analysis carried out in developing nations. In this study, we included these factors in the cost-effectiveness analysis, which may possibly be the cause for the difference found in relation to the current literature. The costs of hospital TB screening may be overestimate. For the hospitalization costs we used the same days spent to detect *M. tuberculosis *in culture (30 days) as proposed by others [19]. The median the days of hospitalization of smear negative pulmonary TB suspects frequently vary from 15 to 25 days in other sites in Brazil, but in this study, it was not measured. The cost-effectiveness per case correctly diagnosed for the AFB smear plus PCR dot-blot strategy was more cost effective than the AFB smear plus culture strategy (ratio of 3.7). The costs for every correctly-diagnosed TB patient were, thus, 3.7 times lower for the AFB smear plus PCR dot-blot than for the AFB smear plus culture, in contrast to previous reports for automated PCR (ratio of 1.8), where this technique was compared to PCR with the smear microscopy routine [11]. The cost-effectiveness per case accurately diagnosed and treated, including treatment of falsely diagnosed cases in AFB smear plus PCR dot-blot, was more cost-effective than for the AFB smear plus culture strategy (ratio of 3.7). The costs for every case accurately diagnosed and treated, including treatment of falsely diagnosed TB patient were thus 3.6 times lower for AFB smear plus PCR dot-blot than for AFB smear plus culture. Furthermore, the cost-effectiveness per case incorrectly diagnosed and not treated, was more cost-effective for AFB smear plus PCR dot-blot than for the AFB smear plus culture strategy (ratio of 3.4). The costs for every case incorrectly diagnosed and not treated TB patient were thus 3.4 times lower for AFB smear plus PCR dot-blot than for AFB smear plus culture.

The over diagnosis due to the low specificity of the *in house *PCR might indeed be a barrier in the decision to invest in these tests. However, in our study, AFB smear plus PCR dot-blot (PCR *in house*) was more cost-effective than AFB smear plus culture, confirming the potential use of *in house *PCR techniques in the diagnostic routine for ruling out of TB diagnosis [12,29-31]. Another reason for the use of the *in house *PCR is that AFB smear plus culture is time consuming and labor intensive. Additionally, the PCR method is patient friendly, reducing costs to patients and, most importantly, PCR is not influenced by the HIV status of the patient [23]

Middle-income countries, like Brazil, have to consider the economic burden of managing false-negative results. The cost of return of all false negative patients to the health services was higher for AFB smear plus culture than for AFB smear plus PCR dot-blot. Some studies have reported the importance of considering the costs of false-negative patients [11,33]. When these costs were associated with total screening costs, the costs for every incorrectly diagnosed and not-treated case were higher in AFB smear plus culture than for the AFB smear plus PCR dot-blot strategy.

The sensitivity analysis, comparing the cost-effectiveness of AFB smear plus culture and AFB smear plus PCR dot-blot, and adjusting different scenarios for TB prevalence, shows that the ratio of cost-effectiveness remains more or less the same in most adjustments. Some gains in cost-effectiveness can be obtained when the sensitivity and specificity of PCR are adjusted to 95%, according to results reported previously that state that a sensitivity of higher than 80% can be a factor in increasing the cost-effectiveness of PCR methods [33].

When reducing the cost of the PCR dot-blot to US$ 10 per test, the cost-effectiveness of the AFB smear plus PCR dot-blot strategy also decrease; such an effect has also been described by others for different methods when the cost of PCR is reduced [11,23,24,33]. It is also expected that cost can be reduced with time and increased utilization [34]. *In house *PCR tests are estimated to cost about US$ 5-10. A price reduction to US$ 6 would adjust the cost effectiveness of PCR to the same level of smear microscopy [33]. The cost-effectiveness of the AFB smear plus PCR dot-blot strategy described in this study was similar to other strategies, when lower TB prevalence made PCR more expensive for diagnosis of PTB [11,33].

## Limitations

a. Since the respiratory specialists were blinded to culture PCR results and laboratory technicians blinded to chest radiograph and clinical predictor results, the study does not mimic the use of PCR dot-blot under field conditions, and, as such, the cost-effectiveness is estimated, not measured.

b. *In-house *PCR results used in this study are not necessarily generalizable to other situations, as *in house *PCR are not standardized and is usually associated with low reproducibility, unless the replicating sites use the exact same *in-house *PCR [5]. Therefore, results obtained by *in house *PCR techniques should be used only by local health service only if the laboratory follows strictly good laboratory practices established by the international guidelines and is certified by regulatory agencies.

c. Twenty-three specimens presented less than 50 CFU in culture were included in the analysis, which is below the detection limit of the PCR test. Partial loss of mycobacterial homogeneity, leading to unequal distribution in the test suspension, may be due to the division of the suspension into three aliquots for use in laboratory tests. Additionally, excluding those samples would increase the in house PCR sensitivity, but it would not represent the routine conditions

d. Stratification of different diagnostic strategy results by HIV status was not performed.

e. Mortality was not measured for either strategy.

f. In the treatment costs analysis, were not included the cost related to: a) the inadequate use of non anti-TB drugs; b) the adverse effects of the inadequate use of anti-TB drugs for non-TB subjects, c) the occurrence of drug-resistant TB, d) the impairment to delay of treatment and of other physical conditions caused by adverse effects of drugs.

g. Isolation use or contact investigation was not included in the analysis

## Conclusions

This study suggests that novel molecular diagnostic tests have the potential to be cost-effective tools in the fight against TB. Our study showed that the use of AFB smear plus PCR dot-blot to diagnose TB, employing the respiratory specimen can be a cost-effective alternative, especially in hospitals of developing nations with a high prevalence of TB and HIV. AFB smear plus PCR dot-blot strategy showed a great improvement in sensitivity, negative predictive value, and offered an improvement for ruling out of pulmonary TB diagnosis. However, inter laboratory and cost-effectiveness studies in other settings are required to evaluate the performance of the PCR dot-blot under field conditions before it may be introduced for routine use.

## Abbreviations List

AFB smear: Acid Fast Bacilli smear; CHC: Community Health Centers; HIV: Human Imunnodeficiency Virus; HIV^+^: HIV seropositive; HIV^-^: HIV seronegative; MOTT: Mycobacterium Other Than Tuberculosis; MTB: *Mycobacterium tuberculosis*; NAA: NucleicAcid Amplification; NPV: Negative Predictive Value; PCR: Polymerase Chain Reaction; PCR-dot-blot: PCR colorimetric dot-blot assay; PPV: Positive Predictive Value; PTB: Pulmonary Tuberculosis; SE: Sensitivity; SP: Specificity; PRH: Parthenon Reference Hospital; TB: Tuberculosis.

## Competing interests

The authors declare that they have no competing interests.

## Authors' contributions

LCS carried out the study, participated in the laboratory tests, participated in data acquisition, performed the statistical analysis and drafted the manuscript; RDS carried out the laboratory tests, data analysis, participated in data acquisition and drafted the manuscript, ARN performed the epidemiological analysis and drafted the paper, MLRR helped design the study, performed the statistical analysis and drafted the paper, CV helped to draft the manuscript, PV performed the statistical analysis and drafted the paper, ALK conceived the study, participated in its design, performed the data analysis, coordination and helped to draft the manuscript. All authors contributed to the interpretation of results and have read and approved the final manuscript.

## Pre-publication history

The pre-publication history for this paper can be accessed here:

http://www.biomedcentral.com/1471-2334/9/216/prepub
